# Genome-Wide Identification of Cyclic Nucleotide-Gated Ion Channel Gene Family in Wheat and Functional Analyses of *TaCNGC14* and *TaCNGC16*

**DOI:** 10.3389/fpls.2018.00018

**Published:** 2018-01-22

**Authors:** Jia Guo, Md Ashraful Islam, Haocheng Lin, Changan Ji, Yinghui Duan, Peng Liu, Qingdong Zeng, Brad Day, Zhensheng Kang, Jun Guo

**Affiliations:** ^1^State Key Laboratory of Crop Stress Biology for Arid Areas, College of Plant Protection, Northwest A&F University, Yangling, China; ^2^Department of Plant, Soil and Microbial Sciences, Michigan State University, East Lansing, MI, United States

**Keywords:** CNGCs, wheat, biotic stress, resistance, *Puccinia striiformis* f. sp. *tritici*

## Abstract

Cyclic nucleotide gated channels (CNGCs) play multifaceted roles in plants, particularly with respect to signaling processes associated with abiotic stress signaling and during host-pathogen interactions. Despite key roles during plant survival and response to environment, little is known about the activity and function of CNGC family in common wheat (*Triticum aestivum* L.), a key stable food around the globe. In this study, we performed a genome-wide identification of CNGC family in wheat and identified a total 47 *TaCNGCs* in wheat, classifying these genes into four major groups (I–IV) with two sub-groups (IVa and IVb). Sequence analysis revealed the presence of several conserved motifs, including a phosphate binding cassette (PBC) and a “hinge” region, both of which have been hypothesized to be critical for the function of wheat CNGCs. During wheat infection with *Pst*, the transcript levels of *TaCNGC14* and *TaCNGC16*, both members of group IVb, showed significant induction during a compatible interaction, while a reduction in gene expression was observed in incompatible interactions. In addition, *TaCNGC14* and *TaCNGC16* mRNA accumulation was significantly influenced by exogenously applied hormones, including abscisic acid (ABA), methyl jasmonate (MeJA), and salicylic acid (SA), suggesting a role in hormone signaling and/or perception. Silencing of *TaCNGC14* and *TaCNGC16* limited *Pst* growth and increased wheat resistance against *Pst*. The results presented herein contribute to our understanding of the wheat CNGC gene family and the mechanism of *TaCNGCs* signaling during wheat-*Pst* interaction.

## Introduction

Calcium ion (Ca^2+^) is an important secondary messenger in modulating multiple signaling pathways. To date, several cation cannels have been reported to mediate Ca^2+^ accumulation in the cytosol including cyclic nucleotide gated channels (CNGCs) (Chin et al., 2009; Ma et al., 2009). Plants use CNGCs for a variety of roles in signal transduction (Talke et al., 2003). As such, plants use this fundamental mechanism to sense and respond to endogenous and environmental stimuli (Jammes et al., 2011). In plant system, CNGCs are cation channels, which are composed of hexa-transmembrane (TM) domains, calmodulin binding domain (CAMB), and cyclic nucleotide-binding domain (CNBD) (Chin et al., 2009; Ma et al., 2009; Zelman et al., 2012; Defalco et al., 2016a). CNBD is the most conserved region found within CNGC proteins, and contains a phosphate binding cassette (PBC) motif and a “hinge” region. PBC binds to the cNMP ligand by catching the sugar and phosphate moieties (Cukkemane et al., 2011), and the hinge region contributes to ligand binding efficacy and selectivity (Young and Krougliak, 2004).

Several plant CNGC genes have been cloned in the past decade including *Arabidopsis* (Köhler and Neuhaus, 2000), barley (Schuurink et al., 1998), and tobacco (Arazi et al., 1999). Additionally, genome-wide analysis of CNGC gene families has been reported in *Arabidopsis* (Mäser et al., 2001), rice (Bridges et al., 2005; Nawaz et al., 2014), Populus (Ward et al., 2009), tomato (Saand et al., 2015a), pear (Chen et al., 2015), and some algae (Zelman et al., 2013). In *Arabidopsis* (Mäser et al., 2001), 20 members of the CNGC gene family have been identified, and 16 in rice (Nawaz et al., 2014); in each, these family members are classified into four groups (I–IV) and two sub-groups (IVa and IVb) based on their phylogenetic relationship (Mäser et al., 2001). Recently, it is hypothesized that CNGC proteins contain a PBC and “hinge” region which identifies only in CNGCs, so these two motifs provide an efficient way to identify plant CNGCs (Zelman et al., 2012, 2013).

Plant CNGCs have been reported to play key roles in response to a variety of abiotic stimuli, including cold stress, salt stress, hormone responses, development, symbiosis, circadian rhythm, and light signaling (Jammes et al., 2011). A few studies suggested that the messenger molecules cAMP and/or cGMP might be responsible for the activation of CNGCs function (Balagué et al., 2003; Chin et al., 2009; Ramanjaneyulu et al., 2010). Plant CNGCs are proved to be involved in some physiological processes including various developmental processes, photo morphogenesis, and tolerance to salt stress (Rubio et al., 2003; Maathuis, 2006), gibberellic acid-induced signaling (Penson et al., 1996), and phytochrome signaling (Bowler et al., 1994). *AtCNGC1* may be involved in Ca^2+^ uptake (Ma et al., 2006), while *AtCNGC3* is required for cellular homeostasis (Gobert et al., 2006). *AtCNGC2, 4, 7, 8, 10, 16*, and *18* have been associated with roles in plant development (Chin et al., 2009; Defalco et al., 2016a).

In addition to the above roles, plant CNGCs have also been demonstrated to be associated with functions in biotic stress signaling (Bowler et al., 1994; Chin et al., 2009; Moeder et al., 2011). Indeed, in *Arabidopsis, AtCNGC2, AtCNGC4, AtCNGC11*, and *AtCNGC12* have been reported to be involved in plant disease resistance; for example, The mutant of *AtCNGC2* (defense no death 1, *dnd1*) exhibited reduced hypersensitive response (HR) response, with enhanced basal resistance to *Pectobacterium carotovorum* (Clough et al., 2000; Ahn, 2007). This mechanism is hypothesized to be associated with *R* gene-associated resistance with partially related to accumulation of salicylic acid (SA) (Yu et al., 1998; Clough et al., 2000; Bock et al., 2006; Genger et al., 2008). *AtCNGC2* provide a model linking Ca^2+^ current to downstream NO production, which leads to HR generation in response to pathogen infection by increasing the cytosolic concentration of Ca^2+^ (Ali et al., 2007; Ma and Berkowitz, 2011). Likewise, a mutant of *AtCNGC4* (defense no death 2, *dnd2*/hypersensitive response-like lesion mimic 1, *hlm1*) showed a similar phenotype to *dnd1*, including a lesion mimic phenotype, high level constitutive expression of pathogenesis-related (PR) genes, and the accumulation of SA (Balagué et al., 2003; Jurkowski et al., 2004). Moreover, the *Arabidopsis* mutant of both of *AtCNGC11* and *AtCNGC12*, also referred to as constitutive expressor of PR gene 22 (*cpr22*), exhibits enhanced resistance to *Hyaloperonospora arabidopsidis* (formerly *Peronospora parasitica*) (Yoshioka et al., 2001, 2006). In tomato, silencing of the *SiCNGC16, 17*, and *18*, which is the ortholog of *AtCNGC2* and *AtCNGC4*, exhibit resistance to *Pythium aphanidermatum* and *Sclerotinia sclerotiorum* while reduces resistance to Tobacco rattle virus (Saand et al., 2015a). In total, these data support a role for CNGCs in plant defense signaling. However, the functions of wheat CNGCs in rust fungi stress responses are largely unknown.

Wheat stripe rust, caused by *Puccinia striiformis* f. sp. *tritici* (*Pst*), is a global threat to wheat production (Wan et al., 2004). In the present study, we identified the CNGC family in common wheat (*Triticum aestivum* L.), one of the most important cereal crops. Our results revealed that the wheat genome contains 47 CNGC genes, and through a comprehensive analysis of this family, we dissected the role of CNGC in wheat resistance signaling between wheat-*Pst* interaction. The present work represents the first comprehensive study in wheat to describe the function of this important gene family.

## Materials and methods

### Identification of CNGC genes in wheat

*In silico*-based methods were used to identify members of CNGC gene family in wheat, including the analysis of 20 *Arabidopsis* CNGC (*AtCNGCs*) genes from the TAIR database^1^ (Mäser et al., 2001), and 16 *Oryza sativa* CNGC (*OsCNGCs*) genes (Nawaz et al., 2014)^2^. Using these sequences, we surveyed the wheat (*T. aestivum cv*. Chinese Spring draft) genome against TGACv1 (Clavijo et al., 2017) using BLASTp^3^. Criteria (E < 10^−5^) were used to ensure the reliability of the protein sequences. Additionally, HMMER 3.0^4^ was used to reduce the candidates *TaCNGC* genes. *AtCNGC* and *OsCNGC* protein sequences were used as a seed file by hmm built to convert input alignments to a profile HMM, and search them against the target candidate sequences database. Finally, all matching sequences did a domain analysis by four programs: Pfam 31.0^5^, PROSITE^6^, SUPERFAMILY 1.75^7^, GENE3D^8^, and CDD^9^. Genes without CNGC-specific CNBD domains and Ion_trans domains were rejected.

### Analysis of TaCNGC predicted proteins features

Prediction of the TaCNGC protein sequences were analyzed by the protein identification and analysis tools on the ExPASy Server^10^ (SIB Bioinformatics Resource Portal) (Gasteiger et al., 2005). Predicted protein length, isoelectric points (PI), molecular weights, instability index, atomic composition, and amino acid composition were predicted. The subcellular localization of the TaCNGC proteins were identified by subloc v1.0 (Hua and Sun, 2001) and ProtComp v9.0^11^.

### Analysis of protein motifs, gene structures, and *Cis*-acting regulatory elements

The protein sequences of 47 TaCNGCs were scanned for conserved motifs using the MEME suite analysis tool version 4.9.1 and MAST motif search tool^12^ with the following parameters: each sequence may contain any number of non-overlapping occurrences of each motif, number of different motifs as 20, range of motif width as 6 to 100. All of the functions of those motifs were analyzed by InterPro and drawn by TBtools software^13^, and the position of the annotated motifs were displayed by the R package “ggplot2”^14^. The structures of *TaCNGC* genes were exhibited using the Gene Structure Display Server (GSDS)^15^. GFF3 files of the wheat genome TGACv1 (Clavijo et al., 2017) was used with default settings. To analyze putative *cis*-elements of *TaCNGC* genes, 1500 bp regions upstream of the mRNA were extracted from TGAVv1 wheat genomic sequences and screened against the Plant-CARE database^16^ (Lescot et al., 2002).

### Phylogenetic analysis of wheat CNGCs

The phylogenetic relationship was inferred with the Maximum Likelihood (ML) method based on LG model (Le and Gascuel, 2008) in MEGA6.0 (Tamura et al., 2013). The midpoint rooted base tree was drawn using Interactive Tree of Life (IToL) Version 3.2.3^17^. Scale bars correspond to 0.1 amino acid substitutions.

### Gene expression analysis

The transcript level of all *TaCNGC* genes were performed by unpublished time series dual RNA-Seq data in our lab. We sequenced two groups of wheat-rust interaction combination, named NIL_R vs. CYR32 and NIL_S vs. CYR32 and selected the time point at 0, 18, 24, 48, 96, and 168 hpi. The wheat cultivar NIL_R (*Yr26*) and NIL_S (*yr26*) were generated by 92R137 (*Yr26* gene donor) backcross with recurrent parent Yangmai 158 for six times and self-cross for four times (BC_6_F_4_) (Wang et al., 2008). A single-spore isolate of *CYR32* was reproduced on seedlings of wheat cultivar Mingxian169. The fresh urediospores were collected and used for inoculating. NIL_S vs. 32R were compatible group (wheat is susceptible to the rust), while NIL_R vs. CYR32 was incompatible group (wheat is resistance to the rust). However, the compatible group and incompatible group were simply named 32S and 32R, respectively. Each sample was sequenced 10 Gb on HiSeq2500 (PE125), and mapped to Chinese spring (TGACv1) (Clavijo et al., 2017) and CYR32 (Zheng et al., 2013) reference.

Depending on the similarity of three homologous of one *TaCNGC* in different sub-genome A, B, and D in wheat, the RPKM of every three homologous were merged, and a heatmap was performed using log_2_(fold change) by the R package “gplots::heatmap.2”^18^.

### Treatments of plants with different stimuli

For chemical treatments, 10-day-old plants were sprayed, separately, with 100 mM abscisic acid (ABA), 100 mM ethylene (ETH), 100 mM methyl jasmonate (MeJA), and 2 mM salicylic acid (SA) (Zhang et al., 2004); each were dissolved in 0.1% (v/v) ethanol. For the mock control, wheat plants were treated with 0.1% (v/v) ethanol. For pathogen inoculation, *Pst* race CYR23 (avirulent) or CYR31 (virulent) was inoculated with the wheat cultivar Suwon 11 following the procedures described previously (Kang et al., 2002). The cultivar Suwon 11, carrying the *YrSu*, shows a typical HR to the *Pst* race CYR23, but is highly susceptible to race CYR31 (Cao et al., 2002). Leaves were gathered at 0, 6, 12, 24, 48, 72, and 120 h post-inoculation (hpi). All samples of these treatments with three independent biological replicates were immediately taken into liquid nitrogen.

### Gene transcriptional level analysis with quantitative real-time PCR

Total RNA was isolated using the TRIzol™ Reagent (Invitrogen, Carlsbad, CA, U.S.A) and digested with DNaseI (TaKaRa, Dalian, China) to eliminate DNA. The RNA was reversed transcription to cDNA by Promega RT-PCR system (Promega, Madison, WI, USA). Quantification of gene transcriptional level was performed with a 7500 Real-Time PCR System (Applied Biosystems, Foster City, CA, U.S.A.). The PCR reactions were conducted according to the procedures and methods as previously described (Duan et al., 2013). A 107-bp fragment of wheat housekeeping gene, *TaEF-1*α (GenBank accession number M90077.1), was amplified as an internal reference for the qRT-PCR analysis, and the data were calculated by the comparative 2^−ΔΔCT^ method (Pfaffl, 2001).

### Virus-induced gene silencing (VIGS) analyses of *TaCNGCs*

The silencing target fragment of *TaCNGC14* and *TaCNGC16* were designed with 249 and 255 bp in 3′ ORF and 5′ UTR, respectively. The *Not*I and *Pac*I restriction sites were used for the primers (Table S1), and the BSMV:γ vector was constructed with those fragments. Capped *in vitro* transcripts were prepared from linearized plasmids containing the tripartite BSMV genome (Petty et al., 1990) using the RiboMAX TM Large-Scale RNA Production System-T7 (Promega, Madison, WI, USA) and the Ribo m7G Cap Analog (Promega, Madison, WI, USA), according to the manufacturer's instructions. Second leaves of two-leaf-stage wheat seedlings were infected with BSMV constructs by rubbing inoculation. After incubation for 24 h in the dark in a humid environment, seedlings were placed in a growth chamber at (25±2)°C. BSMV:TaPDS was used as a positive control (Holzberg et al., 2002). Control plants were treated with 1× Fes buffer (0.1 M glycine, 0.06 M K2HPO4, 1% w/v tetrasodium pyrophosphate, 1% w/v bentonite, and 1% w/v celite, pH 8.5) devoid of BSMV transcripts. The fourth leaf of each plant was inoculated with urediospores of CYR23 or CYR31 at 10 dpi. These leaves were sampled at 0, 24, 48, and 120 hpi for RNA isolation and histological observation. Infection phenotypes of *Pst* were performed at 14 dpi. Absolute quantification by qRT-PCR was used to measuring the biomass changes (Li et al., 2011). The standard curves for wheat and *Pst* were established with the recombinant plasmids carrying either *TaEF* or *PsEF* (Liu et al., 2015). The experiment was done with three replications, and 50 plants were used for each fragment each time.

### Histological observations of fungal infection and host responses

Leaf samples were collected at 24, 48, and 120 hpi with *Pst* and stained as previously described (Wang et al., 2007). Auto-fluorescence of infected mesophyll cells was observed as a necrotic area by epifluorescence microscopy (excitation filter, 485 nm; dichromic mirror, 510 nm; and barrier filter, 520 nm). H_2_O_2_ accumulation was detected by staining with 3,3′-diaminobenzidine (DAB, Amresco, Solon, OH, USA). Wheat germ agglutinin (WGA) conjugated to Alexa 488 (Invitrogen, USA) (10) was used to stain the samples to visualize pathogen structures. The infection sites calculated when the vesicle under a stoma was observed. A minimum of 30 infection sites were examined on each of five randomly selected leaf segments for every treatment. The H_2_O_2_ accumulation, necrotic areas, and hyphal length were observed by Olympus microscope BX-53 (Olympus Cororation, Tokyo, Japan) and calculated by DP-BSW software. Standard deviations and Tukey's test for statistical analysis were performed with the SPSS 16.0 software (SPSS, Inc., Chicago, IL, USA). The relative transcript levels of the pathogenesis related (PR) protein gene *TaPR1* and ROS-related gene *TaCAT1* were analyzed by qRT-PCR in comparison with the control plants in each assay as described above.

### SA quantification

To analyze SA quantification, fresh infection tissue (100–200 mg each sample) was grind to extract SA for HPLC-MS/MS as described (Segarra et al., 2006). MeOH-H_2_O-HOAc (90:9:1, v/v/v) were used as the leaching liquor and MeOH as the mobile phase. The elution gradient in liquid chromatography was carried out with a binary solvent system consisting of 0.05% HOAc in H_2_O (solvent A) and MeOH (solvent B) at a constant flow-rate of 800 μL min^−1^.

## Results

### Identification of *CNGC* genes in wheat genome

To identify CNGC genes in wheat genome (TGACv1), BLAST+ was performed for genes based on the sequence of the 20 Arabidopsis CNGCs (Mäser et al., 2001) and 16 *CNGCs* in rice (Nawaz et al., 2014). Hidden Markov models (profile HMMs) of cNMP_binding domain (PF00027.28) and Ion_trans domain (PF00520.30) were submitted to search against the TGACv1 wheat genome using HMMER3.1. Eighty-one putative genes were found in the TGACv1 protein database except one gene *TaCNGC14A* was assembled by two sequences (AA1199970 and AA2156740). Domain composition analyses using HMMer database indicated that 34 of the 81 candidate sequences carried a AKT/KAT domain, which is annotated as the potassium channel (Shaker type) homologs (Su et al., 2001). Those genes including ion transport and CNBD domain and additional AKT/KAT domain were rejected.

The allohexaploid bread wheat genome is reported that formed via fusion of *T. urartu* (subgenome A)*, Aegilops speltoides* (subgenome B), and *A. tauschii* (subgenome D) genomes before several hundred thousand years ago (Petersen et al., 2006). The A, B, and D sub-genomes contained 60.1–61.3% ratio of genes with orthologs in all the related diploid genomes (International Wheat Genome Sequencing, 2014). Finally, 47 full length *CNGC* genes were identified in wheat genome (Tables S2, S3), including 16, 16, 14 loci in sub-genomes A, B, D with one unknown loci, respectively. The sequences were renamed in ascending order based on the phylogenetic relationship of rice CNGC families (Nawaz et al., 2014). Two genes *TaCNGC1D* and *TaCNGC14D*, were predicted from the genome, both of which were found to have high sequence similarity with genes present in subgenomes A and B. Moreover, some genes lacked the homologous triplet genes (*TaCNGC1, TaCNGC10*, and *TaCNGC13*), and *TaCNGC5* and *TaCNGC7* have duplicate genes named *TaCNGC5/7.1* and *TaCNGC5/7.2*.

### TaCNGC protein features and domain analysis

The TaCNGCs were basic proteins with an average value 9.36 (8.6–9.89) of the isoelectric point (pI). Protein features analysis showed that the size ranging in length of 486 (TaCNGC11B) to 773 (TaCNGC12A) amino acids (aa), averaged of 687 aa, and the average molecular weights are 78.64 kDa (ranging from 55.79 to 88.47 kDa) (Table 1 and Table S3).

**Table 1:** Predicted sequence features of TaCNGC proteins.

**Group**	**Protein ID**	**Length(aa)**	**MW. (kD)**	**pI**	**Sub**.	**NLS pred**	**Domain organization**				
							**Pfam**	**PROSITE**	**CDD**	**Superfamily**	**Gene3D**
Group I	TaCNGC1A	698	80491.44	9.11	P.m	–	1.2	4	5.6	8	9
	TaCNGC2/3A	557	64042.47	9.41	P.m	–	1.2	4	5.6	8	9
	TaCNGC2/3B	501	57700.9	9.35	C.t.m	–	1.2	4	5.6	8	9
	TaCNGC2/3D	694	80164.97	9.39	P.m	–	1.2	4	5.6	8	9
Group II	TaCNGC4A	749	85707.71	9.3	P.m	8(677)	1.2.3	3.4	5.6	8	9
	TaCNGC4B	654	75713.34	8.67	P.m	9(582)	1.2.3	3.4	5.6	8	9
	TaCNGC4D	719	82408.04	9.19	P.m	9(647)	1.2.3	3.4	5.6	8	9
	TaCNGC5.1A	688	79342.9	9.32	P.m	8(620)	1.2	3.4	5.6	8	9
	TaCNGC5.1B	680	78731.42	9.34	P.m	–	1.2	3.4	5.6	8	9
	TaCNGC5.1D	689	79598.24	9.3	P.m	–	1.2	3.4	5.6	8	9
	TaCNGC5.2A	699	81429.93	9.47	P.m	11.5(625)	1.2	3.4	5.6	8	9
	TaCNGC5.2B	698	81040.47	9.63	P.m	16.5(623)	1.2	3.4	5.6	8	9
	TaCNGC5.2D	698	81083.55	9.52	P.m	11.5(624)	1.2	3.4	5.6	8	9
	TaCNGC6A	716	82265.58	9.24	P.m	6(639)	1.2	3.4	5.6	8	9
	TaCNGC6B	716	82233.54	9.15	P.m	6(639)	1.2	3.4	5.6	8	9
	TaCNGC6D	717	82303.53	9.2	P.m	6(639)	1.2	3.4	5.6	8	9
Group III	TaCNGC7.1A	704	81285.82	9.89	P.m	15(652)	1.2	4	5.6	8	9
	TaCNGC7.1B	717	82965.58	9.78	P.m	15(665)	1.2	4	5.6	8	9
	TaCNGC7.1D	702	81123.59	9.89	P.m	15(650)	1.2	4	5.6	8	9
	TaCNGC7.2A	685	79790.91	9.49	P.m	15(634)	1.2	4	5.6	8	9
	TaCNGC7.2B	717	83536.12	9.45	P.m	15(666)	1.2	4	5.7	8	9
	TaCNGC7.2D	685	79804.79	9.48	P.m	15(634)	1.2	4	5.6	8	9
	TaCNGC8A	724	82040.49	8.82	P.m	15(599)	1.2	4	5.6	8	9
	TaCNGC8B	722	81835.35	8.88	P.m	15(602)	1.2	4	5.6	8	9
	TaCNGC8D	725	82108.55	8.82	P.m	15(602)	1.2	4	5.6	8	9
	TaCNGC9A	706	81564.85	9.02	P.m	–	1.2	4	5.6	8	9
	TaCNGC9B	706	81817.22	9.24	P.m	–	1.2	4	5.6	8	9
	TaCNGC9D	706	81918.18	9.09	P.m	–	1.2	4	5.6	8	9
	TaCNGC10A	695	79910.5	9.24	P.m	–	1.2	4	5.6	8	9
	TaCNGC10B	695	79859.34	9.16	P.m	–	1.2	4	5.6	8	9
	TaCNGC10D	695	79840.47	9.24	P.m	–	1.2	4	5.6	8	9
	TaCNGC10.2A	653	74633.71	8.6	P.m	–	1.2	3.4	5.6	8	9
	TaCNGC11B	486	55788.27	9.53	C.t.m	16(415)	1.2	3.4	5.6	8	9
	TaCNGC11D	696	79915.25	9.62	P.m	16(624)	1.2	3.4	5.6	8	9
	TaCNGC11U	695	79581.8	9.55	P.m	16(623)	1.2	3.4	5.6	8	9
Group IVa	TaCNGC12A	773	88467.96	9.27	P.m	9(737)	1.2	4	5.6.7	8	9
	TaCNGC12B	662	75951	9.47	P.m	9(626)	1.2	4	5.6.7	8	9
	TaCNGC12D	565	64599.71	9.44	P.m	9(529)	1.2	4	5.7	8	9
	TaCNGC13B	755	85396.93	9	P.m	–	1.2	3.4	5.7	8	9
Group IVb	TaCNGC14A	720	80688.33	9.34	P.m	6(504)	1.2	4	5.6	8	9
	TaCNGC14B	685	75749.29	9.4	P.m	6(459)	1.2	4	5.6	8	9
	TaCNGC15A	675	75203.25	9.79	nucl	10(42)	1.2	4	5.6	8	9
	TaCNGC15B	672	75289.54	9.86	nucl	10(42)	1.2	4	5.6	8	9
	TaCNGC15D	673	75457.57	9.87	nucl	10(42)	1.2	4	5.6	8	9
	TaCNGC16A	708	79155.56	9.62	E.R./P.m	–	1.2	4	5.6	8	9
	TaCNGC16B	701	78597	9.67	P.m	–	1.2	4	5.6	8	9
	TaCNGC16D	698	78218.57	9.63	P.m	–	1.2	4	5.6	8	9

*MW, Molecular weight; pI, Isoelectric point; P.m, plasma membrane; Cyt, cytoplasm; C.t.m, chloroplast thylakoid membrane; E.R, endoplasmic reticulum; nucl,nucleus; 1, Ion_trans (Ion transport domain); 2, cNMP_binding (Cyclic nucleotide-binding domain); 3, IQ (isoleucine glutamine); 4, CNMP_BINDING_3 (Cyclic nucleotide-binding domain); 5, CAP_ED (Cap family effector domain, which binds cAMP); 6, Ank_2 superfamily; 7, Ion_trans (Ion transport domain); 8, cNMP_bd-like (Cyclic nucleotide-binding-like domain); 9, RmlC (RmlC-like jelly roll fold)*.

The subcellular localization prediction showed that two TaCNGCs (TaCNGC2/3B and TaCNGC11B) are localized in the chloroplast thylakoid membrane (C.t.m), and TaCNGC15a/b/c is localized within the nucleus. The remainder of the TaCNGCs are all predicted to be plasma membrane-localized (TaCNGC16 has the similar score of endoplasmic reticulum and plasma membrane). Indeed, putative nuclear localization signal (NLS) sequences can be found in 30 TaCNGCs (Table 1).

Domain composition analyses using Pfam, Psosite, CDD, Superfamily, and Gene3D confirmed the presence of CNBD/Cyclic Nucleotide-Monophosphate Binding Domain (cNMP, cNMP_binding or cNMP_binding like), Cap Family Effector Domain (CAP_ED), Ank_2 superfamily, RmlC-like jelly roll fold (RmlC), and ion transport domains. In addition, the isoleucine glutamine calmodulin-binding motif was observed to be widely distributed in 17 TaCNGCs (Table 1).

### Gene structure and motif composition analysis

To investigate the gene structure of *TaCNGC* genes, we analyzed the gene structure by the gff3 annotation indicated that most of the *TaCNGCs* have introns (Figure S1). The exon/intron structure exhibited by GSDS found that 6 genes are intronless (*TaCNGC5.1A/B/D* and *TaCNGC5.2A/B/D*) while the numbers of introns for the rest of *TaCNGC* genes varied from 2 to 11. Groups I, II, III, and IVb showed similar features, including 2~6 phase 0 or 1 introns (*TaCNGC5.1, 5.2A/B/D* have 0 introns), while group IVa carrying over 9 introns and all have phase 2 type. It is similar with the *Arabidopsis* and the tomato CNGC genes except the phase type of those are belonging to 1 type (Saand et al., 2015a).

**Figure 1:**
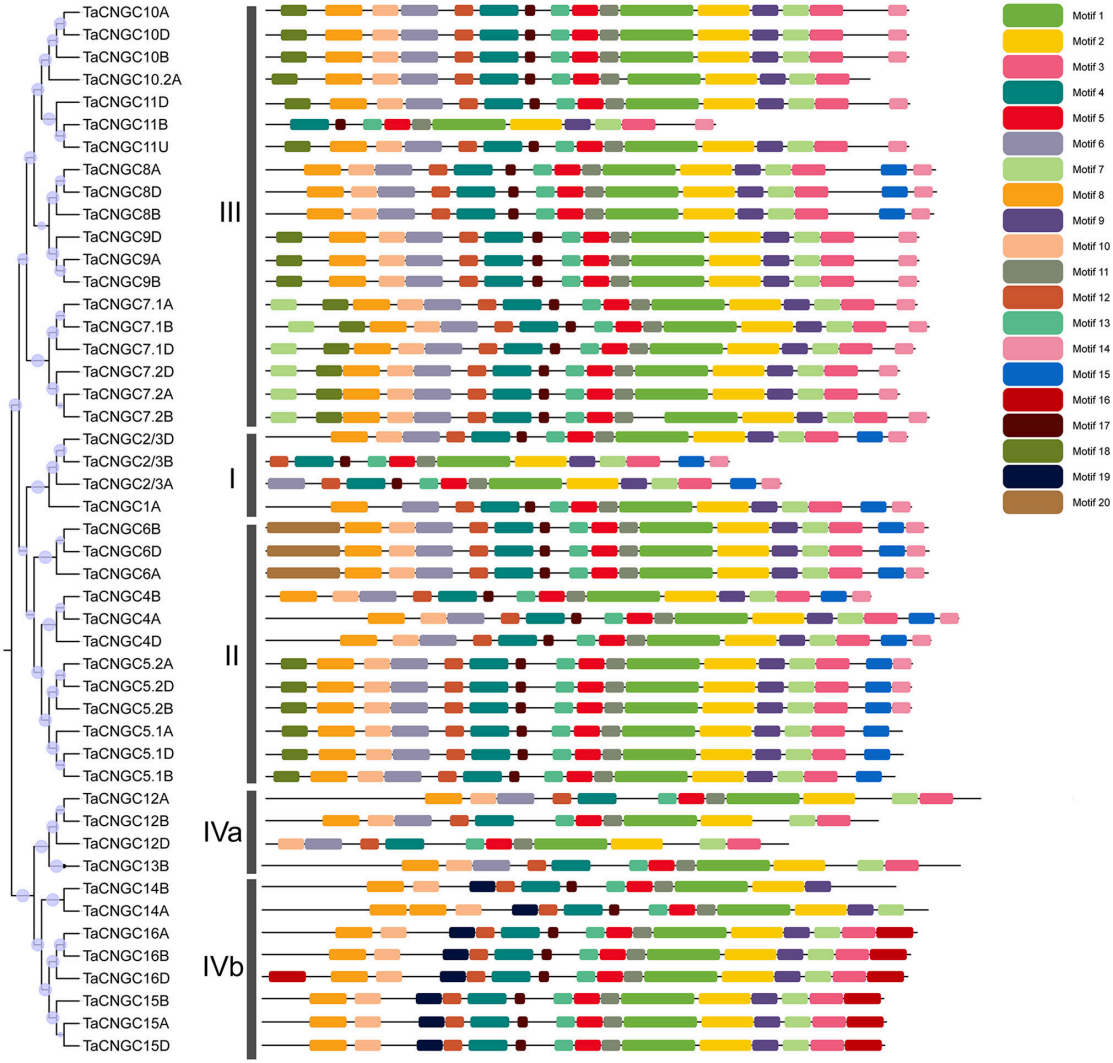
Phylogenetic analysis and conserved motifs in wheat CNGC proteins. The tree was created with bootstrap of 1000 by maximum likelihood (ML) method in MEGA6. The TaCNGCs were divided into four groups with the two sub-group of IVa and Ivb. Model exhibition of motifs composition in TaCNGC amino acid sequences using MAST.

Motif identification queried 20 conserved motifs in the wheat protein by the MAST online software (Figure 1). Six of these motifs were found to be associated with the functionally defined domains. Motif 1 and motif 2 were referred to the cyclic nucleotide-binding domain (CNBD), which was the typical conserved domain in the middle of all the *TaCNGC* genes (Figure S2), and motif 3 was associated with the isoleucine glutamine motif behind the CNB domain. The motif 4, 5, and 6 all belong to ion transport domains in front of the protein, which were interrupted by the non-functional motifs 12, 13, and 17 (Figure S2).

**Figure 2:**
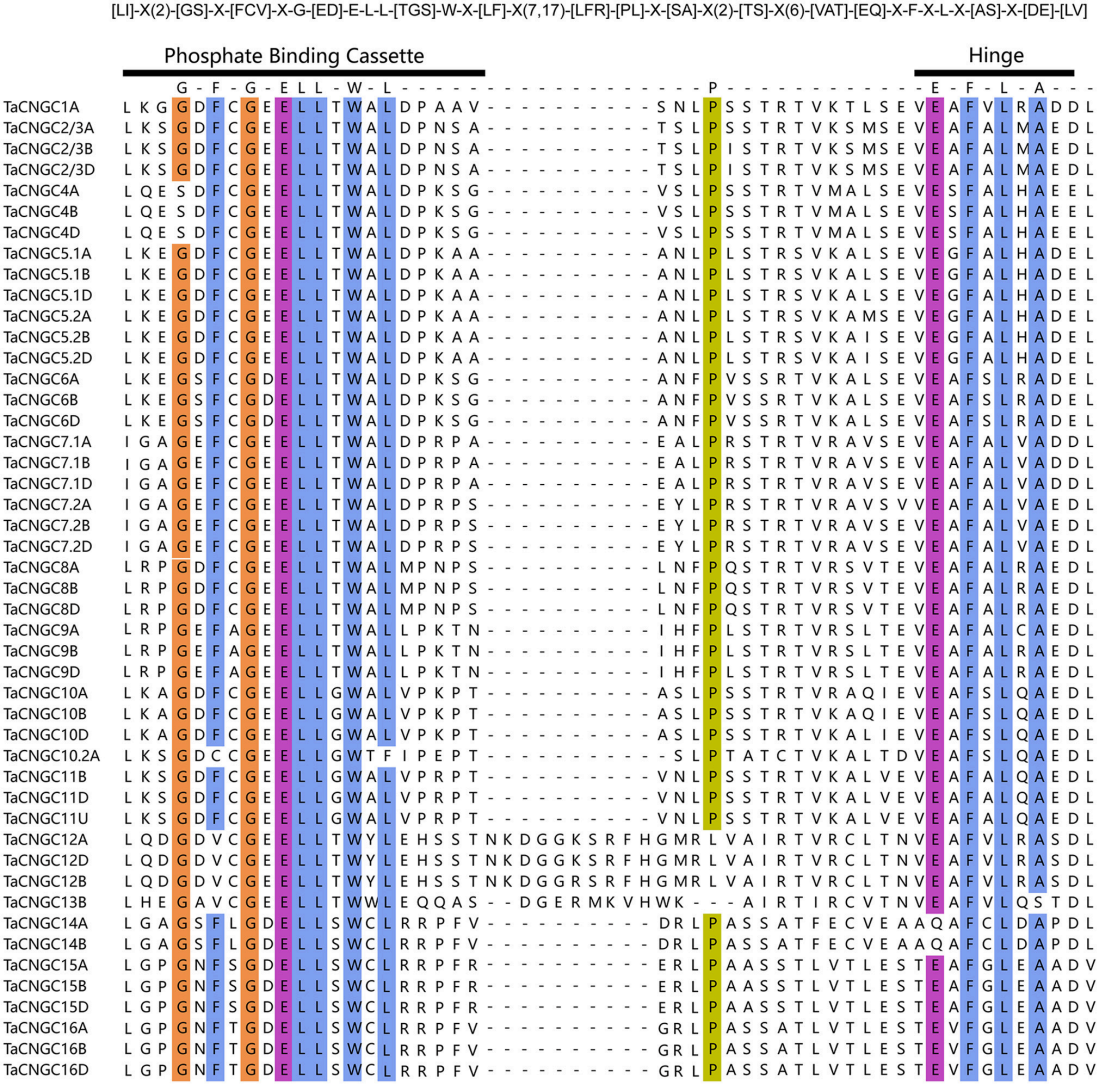
The PBC and the hinge motif within the CNBD of all the TaCNGCs. The wheat CNGC-specific motifs are shown at top. “[]” indicate the various possible of amino acids, “X” could be any amino acid, and “()” represents the number of amino acids. Conserved residues (>90%) were in colorful highlighted among all TaCNGCs, and performed by Ugene program.

Furthermore, PBC and hinge motif were analyzed to find out the relationship of the TaCNGCs and other species' CNGCs. The alignment of the PBC and hinge motif of all the 47 TaCNGCs described as a conserved motif: [LI]-X(2)-[GS]-X-[FCV]-X-G-[ED]-E-L-L-[TGS]-W-X-[LF]-X(7,17)-[LFR]-[PL]-X-[SA]-X(2)-[TS]-X(6)-[VAT]-[EQ]-X-F-X-L-X-[AS]-X-[DE]-[LV] (Figure 2). This result displayed a conserved glycine (G), acidic residue glutamate (E) followed by two aliphatic leucine (L) residues and aromatic tryptophan (W) in PBC motif. In addition, the hinge region takes a conserved aromatic phenylalanine (F) and leucine (L) (Figure 2). Compared this motif with that of plant CNGCs ([LI]-X(2)-[GS]-X-[VFIYS]-X-G-X(0,1)-[DE]-L-[LI]-X-[WN]-X(6,32)-[SA]-X(9)-[VTI]-[EN]-[AG]-F-X-[LI]) (Zelman et al., 2013), demonstrated that TaCNGCs generally fitted the motif of plant CNGC and all the conserved amino acids(G, L, S, E, and F) of the motif of plant CNGC also existed in wheat CNGCs (Zelman et al., 2012, 2013).

### Prediction of *Cis*-acting regulatory elements

Feature of the cis-acting elements were character to obtain preliminary function of the *TaCNGCs*. The 1.5 kb of 5′ upstream non-coding sequences are used to analysis the cis-acting in Plant-CARE database. Those sequences predicted as the promoter sequences revealed that *TaCNGC* genes possess a variety of cis-elements related to various exogenous stimuli, such as ABA, Auxin, MeJA, SA, ETH, and Gibberellin treatment, as well as biotic and abiotic factors (Table S4). Interestingly, we did observe differences in the promoter elements of many of the *TaCNGC* genes, suggesting potentially unique functions. For example, the ETH elements were identified only in *TaCNGC7.2B, TaCNGC8B*, and *TaCNGC16B*, while the CEI element, involved in ABA signaling, was only observed in *TaCNGC13B*. In total, we identified only two ABA responsive, four Auxin responsive, one SA responsive, and one ETH responsive element in a small number of the *TaCNGCs*. These data suggest that the various *TaCNGC* genes are regulated by different stimuli, and perhaps, these unique elements not only specify regulation, but also unique function.

### Phylogenetic relationship analysis

**Figure 3:**
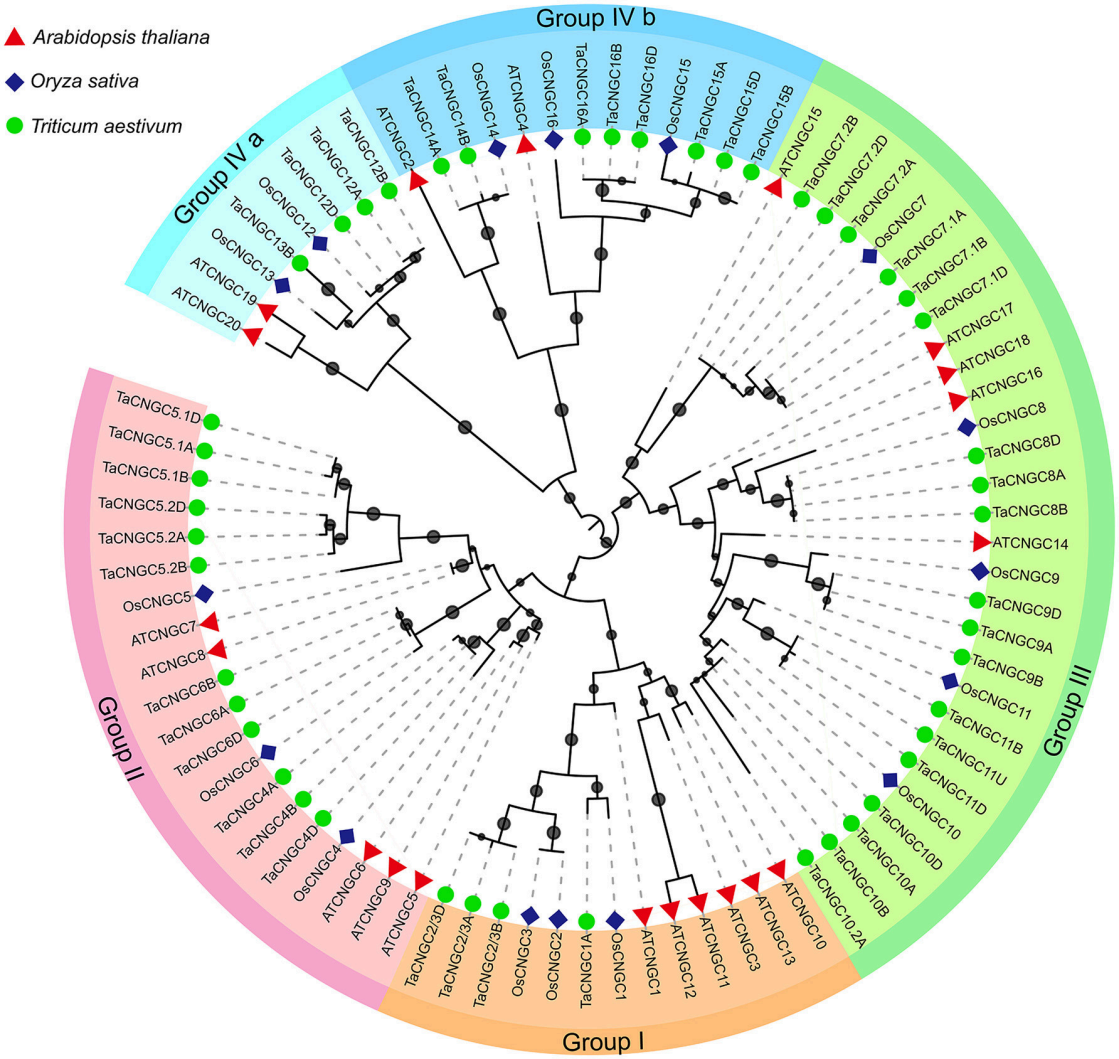
Phylogenetic relationship of TaCNGCs, OsCNGC, and AtCNGC proteins. The maximum likelihood (ML) tree under the Le_Gascuel_2008 model (LG) model was constructed by the MEGA6 software. The midpoint rooted base tree was drawn using Interactive Tree of Life (IToL) Version 3.2.3. Scale bars correspond to 0.1 amino acid substitutions. Different groups were marked by different colors, and the CNGCs from wheat, rice, and Arabidopsis were distinguished with different shapes and colors.

To investigate the relationship among the wheat CNGC proteins, a phylogenetic tree was generated using the available full-length amino acid sequences using the maximum likelihood (ML) method (Figure 1). Among the 47 TaCNGCs, four groups were clustered, similar to that previously described for Arabidopsis CNGCs (Mäser et al., 2001). In addition, a ML phylogenetic tree was constructed to determine the phylogenetic relationship of the CNGC family among rice, wheat and *Arabidopsis*. Twenty AtCNGC proteins (Mäser et al., 2001), 16 OsCNGC proteins (Nawaz et al., 2014), and 47 TaCNGC proteins also gathered into four groups (Figure 3). Especially, the group IV is divided into two sub-groups, named group IVa and IVb. For each group of AtCNGCs and OsCNGCs, wheat homologs existed, and the numbers of the groups are also different. Group I included four from TaCNGCs (TaCNGC1 and TaCNGC2/3A/B/D), six from AtCNGCs (AtCNGC1, 3, 10, 11, 12, and 13) and three from rice CNGCs (OsCNGC1 to OsCNGC3). Similarly, Group II embraces 12 of wheat CNGCs (TaCNGC4A/B/D, TaCNGC5.1, 5.2A/B/D, and TaCNGC6A/B/D), 3 of rice CNGCs (OsCNGC4 to OsCNGC6), and 5 of *Arabidopsis* (AtCNGC5 to AtCNGC9). While group III is the largest, and contained 19 wheat CNGCs (TaCNGC7.1 and 7.2 to TaCNGC10 and 10.2, also contained the all the wheat sub genome A, B, and D), 5 AtCNGCs (AtCNGC14 to AtCNGC18), and 5 OsCNGCs (OsCNGC7 to OsCNGC11). However, four TaCNGCs (TaCNGC12A/B/D and TaCNGC13B), two AtCNGCs (AtCNGC19 and AtCNGC20), and two OsCNGCs (OsCNGC12 and OsCNGC13) were divided into group IVa, while, eight TaCNGCs (TaCNGC14 to TaCNGC16, only TaCNGC14 do not have the D sub-genome genes TaCNGC14D), three rice CNGCs (OsCNGC14 to OsCNGC16), and two AtCNGCs (AtCNGC2 and AtCNGC4) were assigned to group IVb (Figure 3). These wheat *CNGC* genes showed a closer phylogenetic relationship with rice (monocot) than with *Arabidopsis* (dicot). In this respect, the names of the *TaCNGC* genes were assigned based on their respective homologies with the rice CNGCs.

### Expression analysis of *TaCNGCs* in wheat-*Pst* interaction

**Figure 4:**
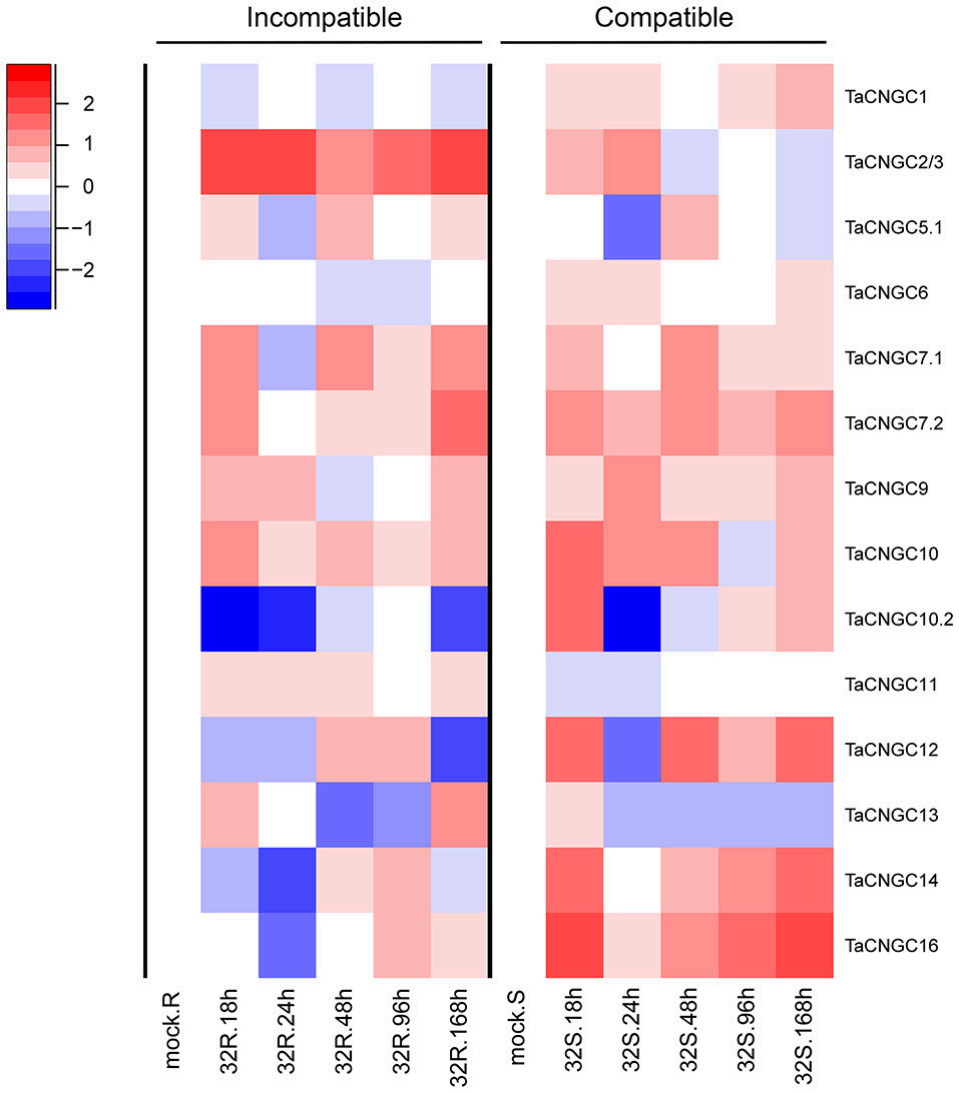
Transcriptional level of *TaCNGCs* in compatible and incompatible interaction between wheat and *Pst*. Expression patterns of *TaCNGCs* were performed with logFC [${\text{log}}_{2}^{\left(\text{foldchange}\right)}$] at 0, 18, 24, 48, 96, and 168 hpi in compatible and incompatible groups by the time series dual RNA-seq data. 32R (right) indicates the incompatible combination, and 32S represents the compatible combination. Blue and red colors indicate down-regulation and up-regulation, respectively. White indicates similar expression patterns as observed with mock treatment.

To determine the roles of *TaCNGCs* in disease resistance, we analyzed the gene transcript levels by the time series dual RNA-seq data in our lab used wheat plant inoculated with *Pst* (unpublished data). FPKM data of *TaCNGCs* were shown in the Table S5 and *TaCNGC4, 5.2, 8*, and *15* were removed by a very low expression level (RPKM < 0.2). The data indicated that *TaCNGC2/3, 7.1, 7.2, 9*, and *10* were up-regulated in most of the time point, and *TaCNGC2/3* showed the highest up-regular in the incompatible group 32R. On the other hand, *TaCNGC1, 6*, and *11* showed a stable expression patterns during all the time point of the compatible and incompatible combination. *TaCNGC10.2* and *TaCNGC12* showed highly similar expression patterns and down-regulated at 12 and 24 hpi in the incompatible group, also in the 24 hpi in the compatible group. There also has one gene, *TaCNGC13*, significantly down-regulated in most of the 32S group and at 48 and 96 hpi in the 32R group. Particularly, two *TaCNGCs* (*TaCNGC14* and *TaCNGC16*) of group IVb were significantly down-regulated in the early stage (18 and 24 hpi) of the incompatible group, while strongly up-regulated in all the stage of compatible group (Figure 4). Our data indicate that *TaCNGC14* and *TaCNGC16* of group IVb may be involved in wheat susceptibility to *Pst*; based on this, these two genes were further characterized, as described below.

### Transcript profiles of *TaCNGC14* and *TaCNGC16* in different stimuli

**Figure 5:**
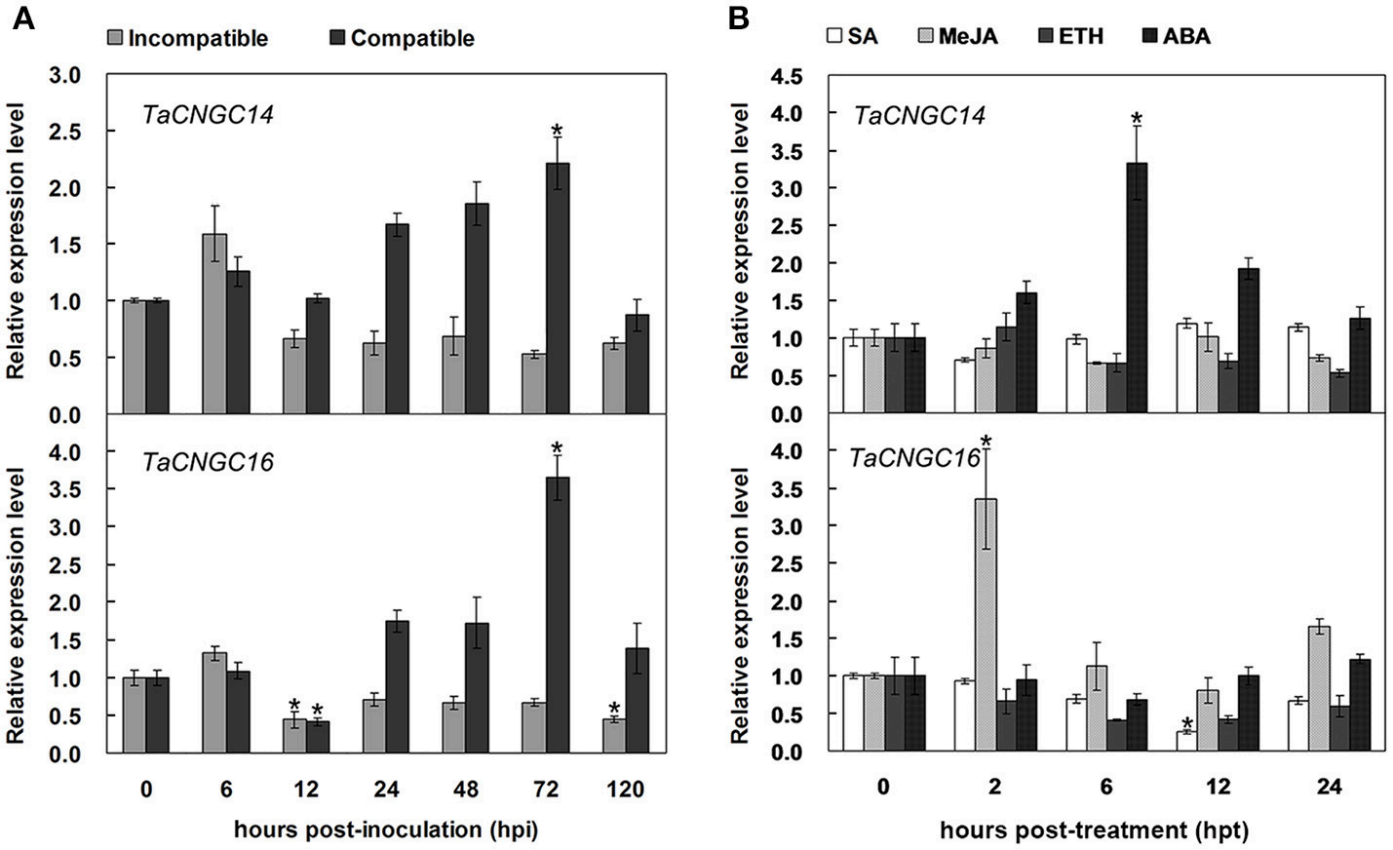
Transcriptional profiles of *TaCNGC14 and TaCNGC16* in wheat leaves during interaction of wheat with *Pst* and exogenous hormone application. **(A)** Leaf samples inoculated *Pst* isolates CYR23 (incompatible interaction) and CYR31 (compatible interaction) were collected at 0, 6, 12, 24, 48, and 120 hpi. **(B)** Four exogenous hormone treatments (SA, MeJA, ETH, and ABA) were sampled with seeding wheat leaves at 0, 2, 6, 12, and 24 hpi. Three biological replicates were calculated by the comparative threshold (2^−ΔΔCT^) method. Asterisks represent significant differences at the same time point using Tukey's HSD test (*P* < 0.05).

To verify the transcript levels of *TaCNGC14* and *TaCNGC16* identified from the RNA-Seq analysis, quantitative real-time PCR (qRT-PCR) analysis was performed (Figure 5A). We used the “Suwon 11 vs. CYR23” group as an incompatible interaction, and the “Suwon 11 vs. CYR31” group as a compatible interaction. *TaCNGC14* showed a significant up-regulation in the compatible interaction at 72 hpi. *TaCNGC16* demonstrated a down-regulation in the incompatible group at 72 hpi while significant up-regulation in the compatible interaction at 12 and 120 hpi. *TaCNGC16* also showed a significant down-regulation during a compatible interaction at 12 hpi. Furthermore, transcript levels of *TaCNGC14* and *TaCNGC16* were determined in seedling wheat leaves under different hormone treatments (SA, MeJA, ETH, and ABA) (Figure 5B). *TaCNGC14* transcripts were significantly increased during ABA treatment, and reached peak (more than three-fold) at 6 hpi. Conversely, *TaCNGC16* mRNA accumulation was up-regulated during MeJA treatment especially at 2 hpi (more than three-fold), while significantly reduced at 12 hpi of SA treatment.

### Silencing of *TaCNGC14* and *TaCNGC16* enhances wheat resistance against *Pst*

**Figure 6:**
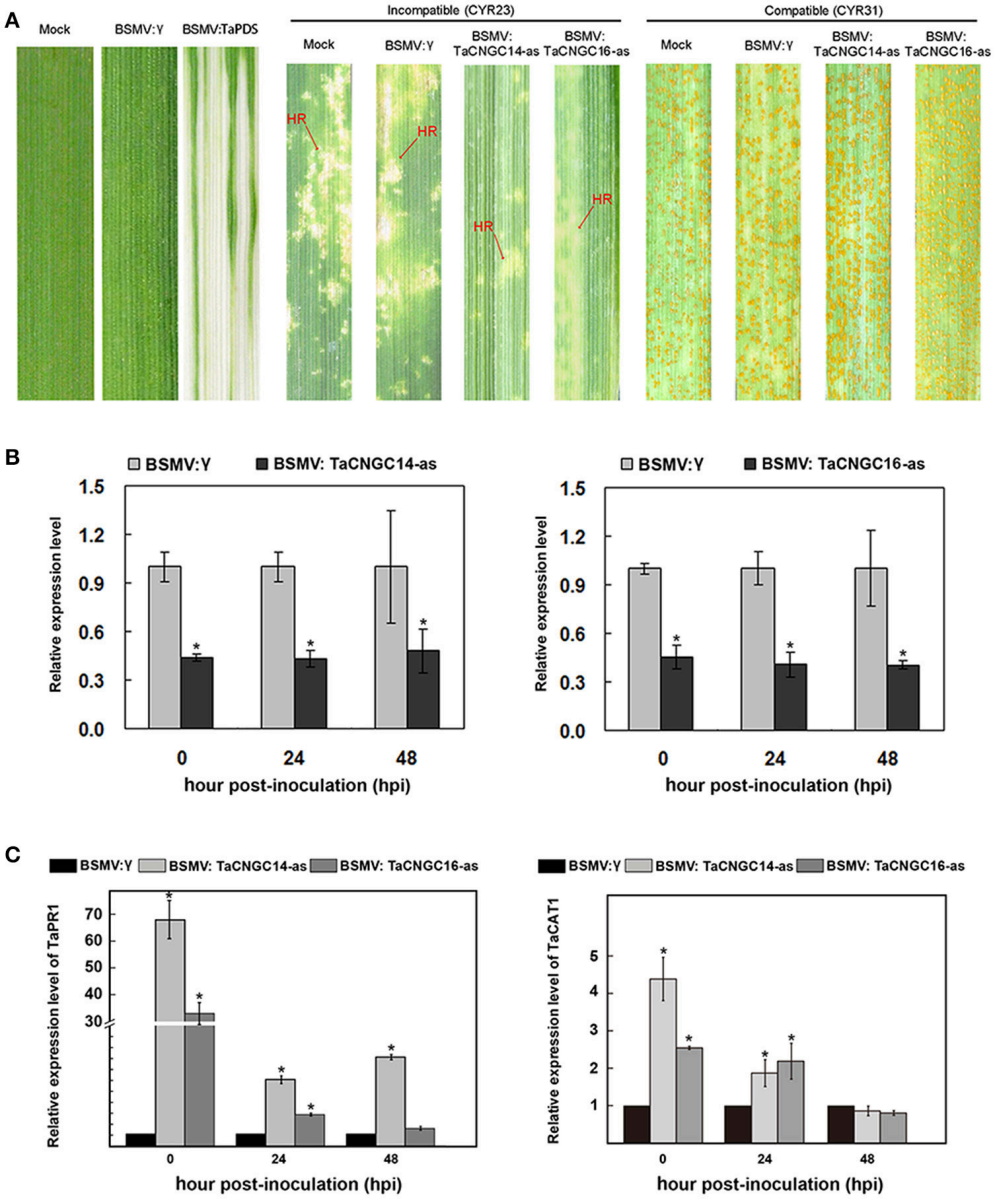
Functional characterization of *TaCNGC14 and TaCNGC16* by BSMV-HIGS. **(A)** BSMV:TaPDS showed photobleaching at 10 dpi; Mock: wheat leaves treated with 1X Fes buffer. Shown are the fourth leaves inoculated with of the avirulent race CYR23, or the virulent race CYR31. Leaves were photographed at 14 dpi. **(B)** Silencing efficiency assessment of two *TaCNGCs* in the fourth leaves of TaCNGCs-knockdown plants inoculated with avirulent race Pst CYR23. **(C)** Transcriptional changes in *PR1* genes and *CAT1* in TaCNGCs-knockdown wheat seedlings. Three biological replicates were calculated by the comparative threshold (2^−ΔΔCT^) method. Asterisks represent significant differences at the same time point by Tukey's HSD test (*P* < 0.05). HR, hypersensitive reaction.

To uncover the function of *TaCNGC14* and *TaCNGC16* during the interaction between wheat and *Pst*, the barley stripe mosaic virus (BSMV)-mediated virus-induced gene silencing (BSMV-VIGS), an effective reverse genetics tool was used (Holzberg et al., 2002; Scofield et al., 2005). Special fragments were designed to knock down the two *TaCNGC* genes using primers specified in Table S1. All of the BSMV-inoculated plants displayed mild chlorotic mosaic symptoms at 10 dpi (days post-inoculation), but they had no obvious defects in further leaf growth, while leaves inoculated with BSMV:*TaPDS* showed photobleaching (Figure 6A), indicating BSMV induced gene silencing system functions well. Compared with BSMV:γ-infected leaves, typical hypersensitive response (HR) was decreased in the *TaCNGCs* silenced plants by inoculating CYR23, while also exhibited normal disease development with CYR31 (Figure 6A). To determine the efficiency of VIGS, qRT-PCR was performed to examine the relative transcript levels of *TaCNGC14* and *TaCNGC16* in the fourth leaves of infected plants. Compared with control inoculations, transcript levels of *TaCNGC14* knockdown plants were reduced by 56, 57, and 52% at 0, 24, and 48 hpi, and *TaCNGC16* knockdown plants also showed a stable efficiency by reducing to 55, 59, and 60% at at 0, 24, and 48 hpi with CYR23, respectively (Figure 6B). Furthermore, the degree of silencing at 5 and 14 dpi was also performed, respectively, and found the transcript levels of *TaCNGC14* and *TaCNGC16* were reduced by 46.1 and 45.8% at 5 dpi and 14.4 and 18.2% at 14 dpi (Figure S4A). Additionally, knocking down *TaCNGC14* and *TaCNGC16* significantly increased the transcript level of *TaPR1* (*TaPR1-13*, GenBank: KR351308.1) at 0, 24, 48 hpi, and increased the mRNA levels of *TaCAT1* ~two-to-three-fold at 0 hpi and 24 hpi (Figure 6C). Meanwhile, *TaPR1.1* (*TaPR1-3*, GenBank: HQ541963.1) was induced in both *TaCNGC14* and *TaCNGC16* knocked-down plant, While, *TaPR1.2* (*TaPR1-20*, GenBank: HQ541980) did not show significate change during the silencing experiment (Figures S4C,D).

**Figure 7:**
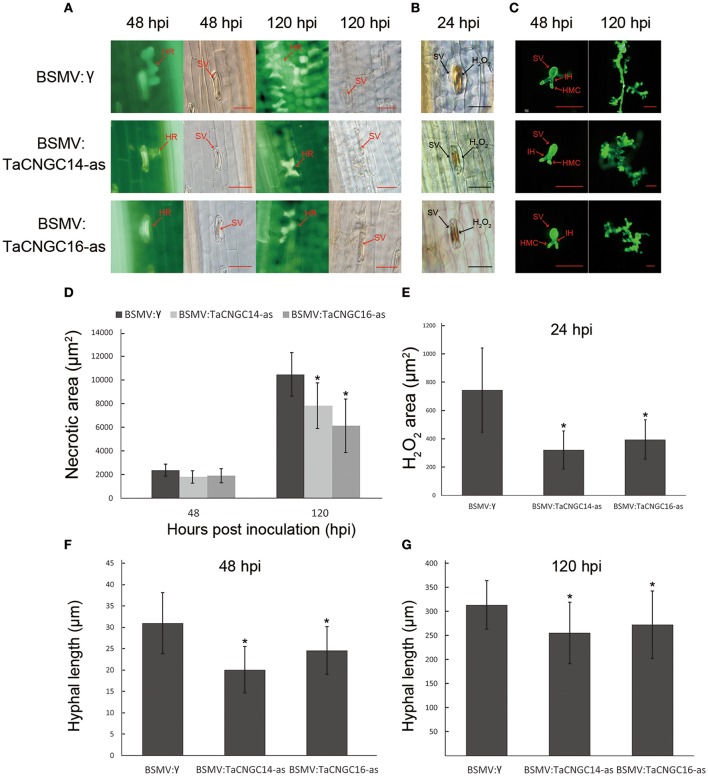
Histological observation of HR, H_2_O_2_ area, and fungi development. **(A)** The silencing leaves were inoculated with CYR23, and the necrotic area necrotic mesophyll cell around an infection site was performed during 48 and 120 hpi by epifluorescence. Mock was treatment with BSMV:γ. **(B)** H_2_O_2_ accumulation was counts at 24 hpi around the infect area by staining with DAB. **(C)** Wheat germ agglutinin (WGA) was used to stain the leaves to visualize pathogen. **(D)** Necrotic area was measured at 48 and 120 hpi. **(E)** H_2_O_2_ area was measured at 24 hpi. **(F–G)** Hyphal lengths were measured at 48 hpi **(F)** and 120 hpi **(G)**. Asterisks represent a significant differences (*P* < 0.05) from BSMV:γ by the Tukey's HSD test. HR, hypersensitive reaction; SV, sub-stomatal vesicle; IH, primary hyphae; HMC, haustorial mother cell. Those data were collected from 30 infection sites. Hpi, hours post-inoculation; Bar, 50 μm.

To observe the disease phenotype in plants inoculated with *Pst*, we examined the infection site of fourth leaves under the microscope. For the *TaCNGC14*- and *TaCNGC16*-silenced plants, the necrotic area was significantly decreased at 48 and 120 hpi (*P* < 0.05) compared to that of control leaves at (Figures 7A,D). Additionally, H_2_O_2_ accumulation at the site of infections were also reduced early time point (24 hpi) (Figures 7B,E), suggesting a role in the early stages of resistance to infection. In addition, the hyphal length of *Pst* were also decreased at 48 and 120 hpi (Figures 7C,F). Fungal and wheat biomass ratio measured via total DNA content at 14 dpi by absolute quantification using the internal reference genes *PsEF* and *TaEF*, respectively. In incompatible group, the *Pst*/wheat ratio was 0.52, 0.47, and 0.48, while it was 0.86, 0.89, and 0.85 in the compatible group in BSMV:γ, BSMV:TaCNGC14, and BSMV:TaCNGC16 plants, respectively (Figure S4B).

In summary, these results indicate that *TaCNGC14* and *TaCNGC16* can be efficiently silenced by the BSMV, and knockdown of the *TaCNGC14* and *TaCNGC16* limited *Pst* growth and increased the plant resistance.

## Discussion

### *TaCNGC* gene family in wheat genome

Cyclic nucleotide-gated channels (CNGCs) gene families from several plant species have been identified and characterized as a result of whole genome sequencing approaches (Mäser et al., 2001; Zelman et al., 2013; Nawaz et al., 2014; Chen et al., 2015; Saand et al., 2015a). From these approaches, it has been demonstrated that the total number of *CNGC* gene families showed large differences in different plants. For example, 20, 16, 21, and 18 *CNGC* genes were identified in *Arabidopsis* (Mäser et al., 2001), rice (Nawaz et al., 2014), pear (Chen et al., 2015), and tomato (Saand et al., 2015a), respectively. In this study, we identified the CNGC family in common wheat (*T. aestivum* L.), one of the most important cereal crops grown around the world for human consumption (Gustafson et al., 2009). Because wheat possess three sub-genomes (A, B, and D), we identified a large number wheat CNGC proteins (16, 16, 14 loci in sub-genomes A, B, D, respectively, with one found in an unknown sub-genome) by bioinformatics analyses (Table S2). Interestingly, previous relevant experimental evidence indicates that most of plant CNGCs are localized to the plasma membrane (Defalco et al., 2016a), while *AtCNGC19* & *20* are components of vacuole membranes (Yuen and Christopher, 2013) and *MtCNGC15a/b/c* (the ortholog of *TaCNGC7.1A/B/D* and *7.2A/B/D*) are the first isoforms found to be localized to the nucleus (Charpentier et al., 2016). In this study, the prediction of *TaCNGC15A/B/D* also located in nucleus and most of *TaCNGCs* (30 of 47 containing a nuclear localization signal (NLS)). The real localization of *TaCNGCs* need to be further analyzed.

Phylogenetic analysis divided *TaCNGC* genes into four groups (group IV also contained two sub-group) (Figures 1, 3), with the similar clusters to rice (Nawaz et al., 2014) and *Arabidopsis* CNGCs (Mäser et al., 2001) (Figure 3). However, CNGC genes exhibit different putative relationships among wheat (monocot), rice (monocot), and *Arabidopsis* (dicot). All 47 *TaCNGCs* showed a closer relationship with the 16 *OsCNGCs*, while less with the *Arabidopsis CNGCs* (Figure 3). Most *Arabidopsis* CNGCs show divergence with the other two taxa. For example, AtCNGC19 and AtCNGC20 were separated from OsCNGC13 and TaCNGC13B branch and OsCNGC12 and TaCNGC12A/B/D branch. The results suggest that the duplication of *CNGC* genes in wheat occurred after speciation and after divergence of angiosperms into monocots and dicots (Nawaz et al., 2014; Saand et al., 2015b). However, AtCNGC2, TaCNGC14A/B, and OsCNGC14 in Group IVb and AtCNGC15, TaCNGC7.1A/B/D, 7.2A/B/D, and OsCNGC7 in Group III are different from other clusters. These data suggest that these homologous genes have already evolved before this separation. Interestingly, we observed that only two rice *CNGC* genes (*OsCNGC2/3*) possessed predicted homologs in wheat (i.e., *TaCNGC2/3A, B*, and *D*), while *OsCNGC5, 7*, and *10* have two groups of wheat homologous genes, named.*1* and.*2* (*TaCNGC10.2* only contained the B sub-genome *TaCNGC10.2B*). Similarly, no homologous genes were identified for *TaCNGC13B* and *TaCNGC10.2B* in wheat, suggesting that these two genes are specific to *A. speltoides* (subgenome B). Moreover, the different family numbers indicated that gene duplication and gene losses play an important role during evolution of a gene family to create new genes and different functions (Chauve et al., 2008). In summary, gene duplications and gene losses within the wheat CNGC family suggest that they have different functions as compared to *OsCNGCs*. Thus, the identification of the wheat CNGC family provides a framework for determining the evolutionary relationship amongst the broader plant CNGC family.

### Functions of *TaCNGCs* in response to *Pst* infection

CNGCs are involved in discrete signaling pathways associated with the regulation of various stress signaling processes, including salt tolerance, drought tolerance, cold tolerance, plant nutrition and calcium homeostasis, and response to pathogens (Defalco et al., 2016b; Jha et al., 2016). However, little is known about the function of *TaCNGCs* under biotic stress. In our study, we focused on determining the roles of *TaCNGCs* in disease resistance; specifically, using the wheat-*Pst* pathosystem. As shown in Figure 4, many of the *TaCNGCs* revealed either up- or down-regulated expressions levels during the incompatible and compatible interaction between wheat and *Pst* (Figure 4), indicating that some of *TaCNGCs* play a potential role in wheat resistance against pathogen. According to the transcriptional profile (Figure 4), *TaCNGC2/3*, the ortholog of *AtCNGC11* and *12*, showed highest up-regulation in the incompatible interactions with the avirulent rust fungi. In *Arabidopsis*, the mutant of both of those two genes called constitutive expressor of *PR* gene 22 (*cpr22*) (Yoshioka et al., 2001) generating a novel chimeric *AtCNGC11/12* (Yoshioka et al., 2006), which exhibits spontaneous lesion formation, SA accumulation, and *PR* gene expression (Yoshioka et al., 2001). In addition, there is also a report that *cpr22* mutants display altered ABA-related phenotypes (Mosher et al., 2010), suggesting that *TaCNGC2/3* may also be involved in pathogen resistance.

*TaCNGC14* and *TaCNGC16* displayed a similar expression pattern, including the down-regulation of mRNA accumulation, in the early stages of the incompatible interaction with an avirulent isolate of *Pst*. Conversely, *TaCNGC14* and *TaCNGC16* were observed to be up-regulated in the compatible group with a virulent strain of *Pst*. Taken together, these data support the hypothesis that these two genes play a negative role in wheat resistance against pathogens. The different stimuli of different hormone treatments showed that *TaCNGC14*, the ortholog of *AtCNGC2*, was induced by the ABA while *TaCNGC16*, the ortholog of *AtCNGC4*, was induced by MeJA and repressed by SA. Interestingly, *TaCNGC16* showed a down-regulation in compatible interaction and SA treatment at 12 hpi, suggesting that *TaCNGC16* may be suppressed by the endogenous SA signal while more evidence need to be further researched. This profile exhibited similar characteristics with *AtCNGC2* and *AtCNGC4* following MeJA treatment; however, *AtCNGC2* was also repressed by SA treatment (Moeder et al., 2011). SA signaling is often effective against biotrophic pathogens, whereas MeJA/ETH signaling is required for effective resistance to necrotrophic pathogens (Glick, 2005). It is reported that pearl millet shown resistance to a virulent isolate of rust, *Puccinia substriata*, during SA treatment, whereas MeJA did not significantly influence infection level (Crampton et al., 2009). These results suggest that SA-mediated signaling pathway is involved in rust resistance. ABA not only plays a role in a diversity of growth and physiological pathways, including abiotic stress responses (Finkelstein et al., 2002), but also has been identified as a crucial regulator of biotic stress response signaling (Ton et al., 2009). Our results suggest that *TaCNGC14* maybe participate in ABA-mediated signaling pathway in wheat resistance to *Pst*, and *TaCNGC16* may be involved in SA-mediated signaling pathway in wheat-*Pst* interaction.

Virus-induced gene silencing (VIGS) mediated by the barley stripe mosaic virus (BSMV) has been recognized as a rapid and effective reverse genetics approach in barley and wheat (Scofield et al., 2005; Senthil-Kumar and Mysore, 2011). Knocking down *TaCNGC14* and *TaCNGC16* by VIGS showed that the area of HR was decreased in the incompatible interaction and the growth of *Pst* was limited. The results are similar with that they have found before in *Arabidopsis* which the loss of function mutant for *AtCNGC2, dnd1*, and for *AtCNGC4, dnd2/hlm1* shows alterations in some phenotypes including the responses to avirulent pathogens, such as impaired HR, accumulate SA, and induced the constitutive expression level of PR protein (Yu et al., 1998; Clough et al., 2000; Balagué et al., 2003; Jurkowski et al., 2004; Genger et al., 2008). Furthermore, the work of supply with exogenous nitric oxide (NO) restored HR in the *dnd1* plant, indicating that NO is essential for HR development, suggested that CNGC-dependent cytosolic Ca^2+^ increase is involved in the PAMP-induced nitric oxide (NO) production (Ali et al., 2007; Ma et al., 2009). The similar phenotype between *TaCNGC14/16* and *AtCNGC2/4* suggested that the *TaCNGC14* and *TaCNGC16* are also involved in the NO signaling pathway to affect the HR development. In addition, the differences in transcript levels of three *TaPR1* genes indicate that *TaPR1.1* and *TaPR1.2* may not be the best marker for the defense response, and *TaPR1.2* expression was unchanged by activators of SAR (such as SA) (Lu et al., 2011; Haque et al., 2014). However, previous studies demonstrated that *TaPR1-13* was significantly during pathogen infection (Fu et al., 2014; Zhu et al., 2017); our results herein support this, revealing that TaPR1-13 is likely involved with signaling during pathogen infection.

After SA treatment, the transcript level of *TaCNGC14* showed no significant change whereas that of its ortholog AtCNGC2 in Arabidopsis exhibited down-regulation. Then we measured the SA level in the *TaCNGC14* knock-down plants, in which showed significant up-regulation (Figure S3), indicating that *TaCNGC14* also have similar function with *AtCNGC2* in SA pathway. However, whether similar mechanism occurs to *TaCNGC14* and *TaCNGC16* in response to avirulent *Pst* and the mechanism of no change in the virulent *Pst* should to be studied further. Taken together, our result indicated that *TaCNGC14* and *TaCNGC16* were involved in response to avirulent *Pst* (with the AVR-R gene partner) and associated with different signal pathways. Like the function of *cpr22* (Mosher et al., 2010), *TaCNGC14* may cause some crosstalk between SA and ABA signaling pathway in wheat.

## Conclusion

In summary, 47 CNGC genes were comprehensively identified from the wheat genome (TGACv1) with 16, 16, 14 members of the CNGC family located in sub-genome A, B, D with one unknown the sub-genome, respectively. *TaCNGC* gene family numbers contain the Ion_trans domain and CNBD domain including a PBC and a “hinge” region described as a stringent motif: [LI]-X(2)-[GS]-X-[FCV]-X-G-[ED]-E-L-L-[TGS]-W-X-[LF]-X(7,17)-[LFR]-[PL]-X-[SA]-X(2)-[TS]-X(6)-[VAT]-[EQ]-X-F-X-L-X-[AS]-X-[DE]-[LV]. Moreover, we found that *TaCNGC14* and *TaCNGC16* showed differential expression between wheat-*Pst* compatible and incompatible interactions, suggesting that *TaCNGC14* and *TaCNGC16* play a negative role in in wheat resistance against pathogens. Furthermore, *TaCNGC14* and *TaCNGC16* exhibited significant response to different hormone stimuli. In addition, silencing of *TaCNGC14* and *TaCNGC16* reduced the HR, while limited the growth of *Pst* and increased the plant resistance. Collectively, the study of the *TaCNGC* gene family in wheat genome provides the comprehensive overview between wheat and *Pst* interaction and make a prospect to further elucidate of the wheat-*Pst* interaction mechanism and the function of plant CNGCs.

## Author contributions

JuG and ZK designed the experiment. JiG, HL, CJ, and QZ conducted the bioinformatics and phylogenetic analysis. YD and MI performed the gene expression and VIGS experiments. JiG, PL, MI, BD, JuG, and ZK wrote the manuscript.

### Conflict of interest statement

The authors declare that the research was conducted in the absence of any commercial or financial relationships that could be construed as a potential conflict of interest.

## Footnotes

^1^http://www.Arabidopsis.org/

^2^http://rice.plantbiology.msu.edu/

^3^http://blast.ncbi.nlm.nih.gov/Blast.cgi

^4^http://www.hmmer.org/

^5^http://pfam.xfam.org/

^6^http://prosite.expasy.org/

^7^http://www.supfam.org/SUPERFAMILY/

^8^http://gene3d.biochem.ucl.ac.uk/

^9^https://www.ncbi.nlm.nih.gov/cdd/

^10^http://www.expasy.org/

^11^http://www.softberry.com/berry.phtml

^12^http://meme-suite.org/tools/meme

^13^https://github.com/CJ-Chen/TBtools

^14^http://ggplot2.tidyverse.org

^15^http://gsds.cbi.pku.edu.cn/

^16^http://bioinformatics.psb.ugent.be/webtools/plantcare/html/

^17^http://itol.embl.de/

^18^https://CRAN.R-project.org/package=gplots

## Supplementary material

Figure S1 Gene structure of 47 *TaCNGC* genes. Yellow boxes represent exons, black lines represent introns, and blue boxes denote the UTR region. Numbers above the introns means the phase 0, 1, and 2.

Figure S2 Motif component and position in the *TaCNGC* genes. Functional motifs were presented on the right using MEME, and clustered based on function. The position of those motifs performed by the box plots, and x-axis indicated the length of all the *TaCNGC* genes (aa).

Figure S3 Quantification of SA level in *TaCNGC14* silencing plants. Silencing leaves which inoculated with CYR23 were isolated from the fourth leaves, BSMV:γ plant is as a control. Results are shown as means ± SE of three biological replications.

Figure S4 qRT-PCR analysis of *TaCNGC14* and *TaCNGC16* silencing plants. **(A)** Silencing efficiency assessment of two *TaCNGCs* in the fourth leaves of *TaCNGCs*-knockdown plants inoculated with avirulent race *Pst* CYR23 at 5 and 14 dpi. **(B)** Fungal and wheat biomass ratio measured via total DNA content at 14 dpi by absolute quantification using the internal reference genes *PsEF* and *TaEF*, respectively. **(C,D)** Transcriptional changes in *PR1.1* genes and *PR1.2* in *TaCNGCs*-knockdown wheat seedlings.

Table S1 Gene ID and location of the *TaCNGCs*.

Table S2 TaCNGCs sequence analysis.

Table S3 cis-acting element of *TaCNGCs*.

Table S4-1 FPKM of all the *TaCNGCs* in RNA-seq data.

Table S4-2 COUNTs of all the *TaCNGCs* in RNA-seq data.

Table S5 Primers used in this study.
