# Supplementary figures and images for: Genome-Wide Identification of Cyclic Nucleotide-Gated Ion Channel Gene Family in Wheat and Functional Analyses of TaCNGC14 and TaCNGC16
Source: Front Plant Sci. 2018 Jan 22;9:18. doi: 10.3389/fpls.2018.00018 (PMC5786745; doi:10.3389/fpls.2018.00018)

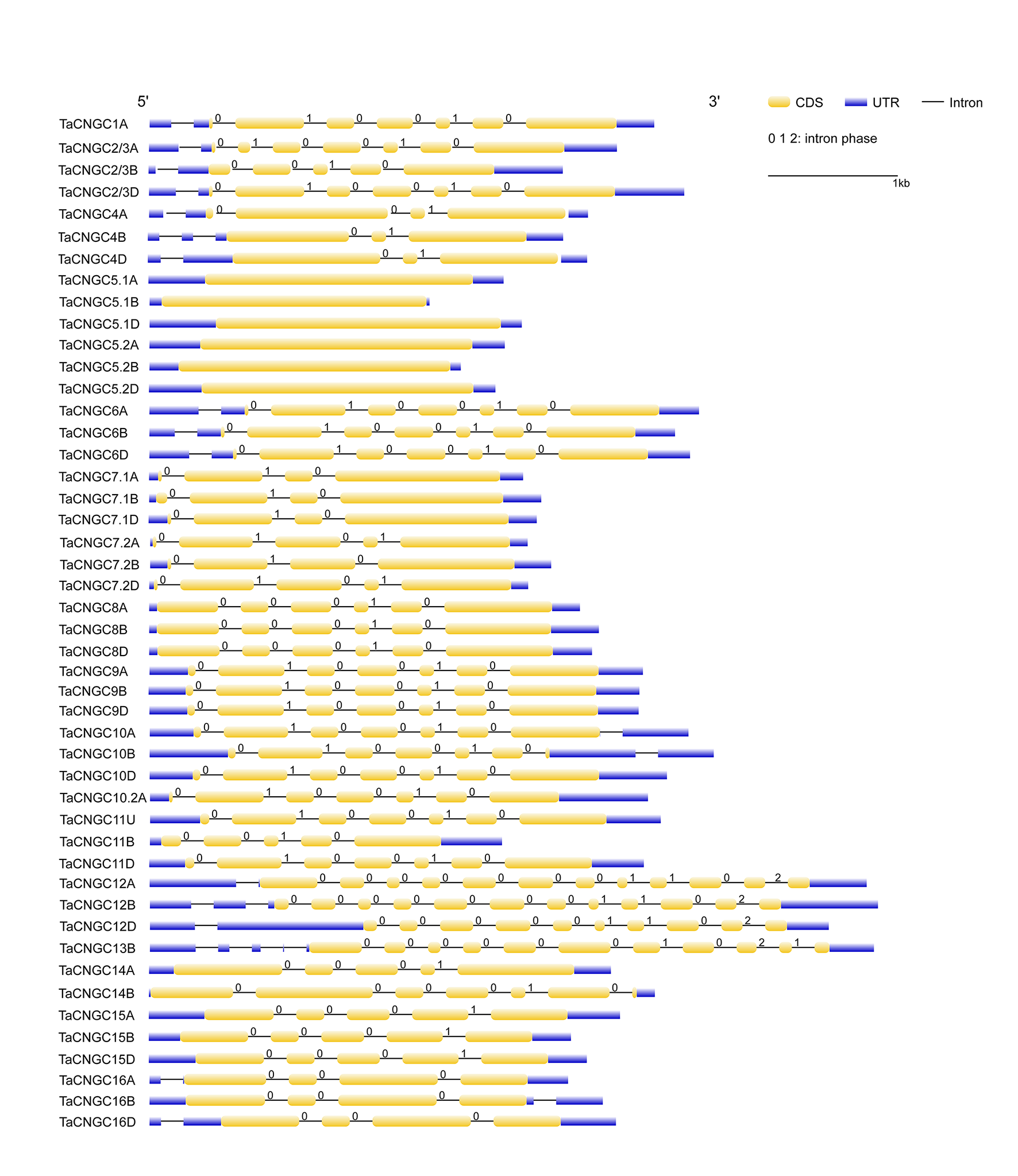

Supplement: Figure S1 — Gene structure of 47 TaCNGC genes. Yellow boxes represent exons, black lines represent introns, and blue boxes denote the UTR region. Numbers above the introns means the phase 0, 1, and 2. [file Image1.TIF]

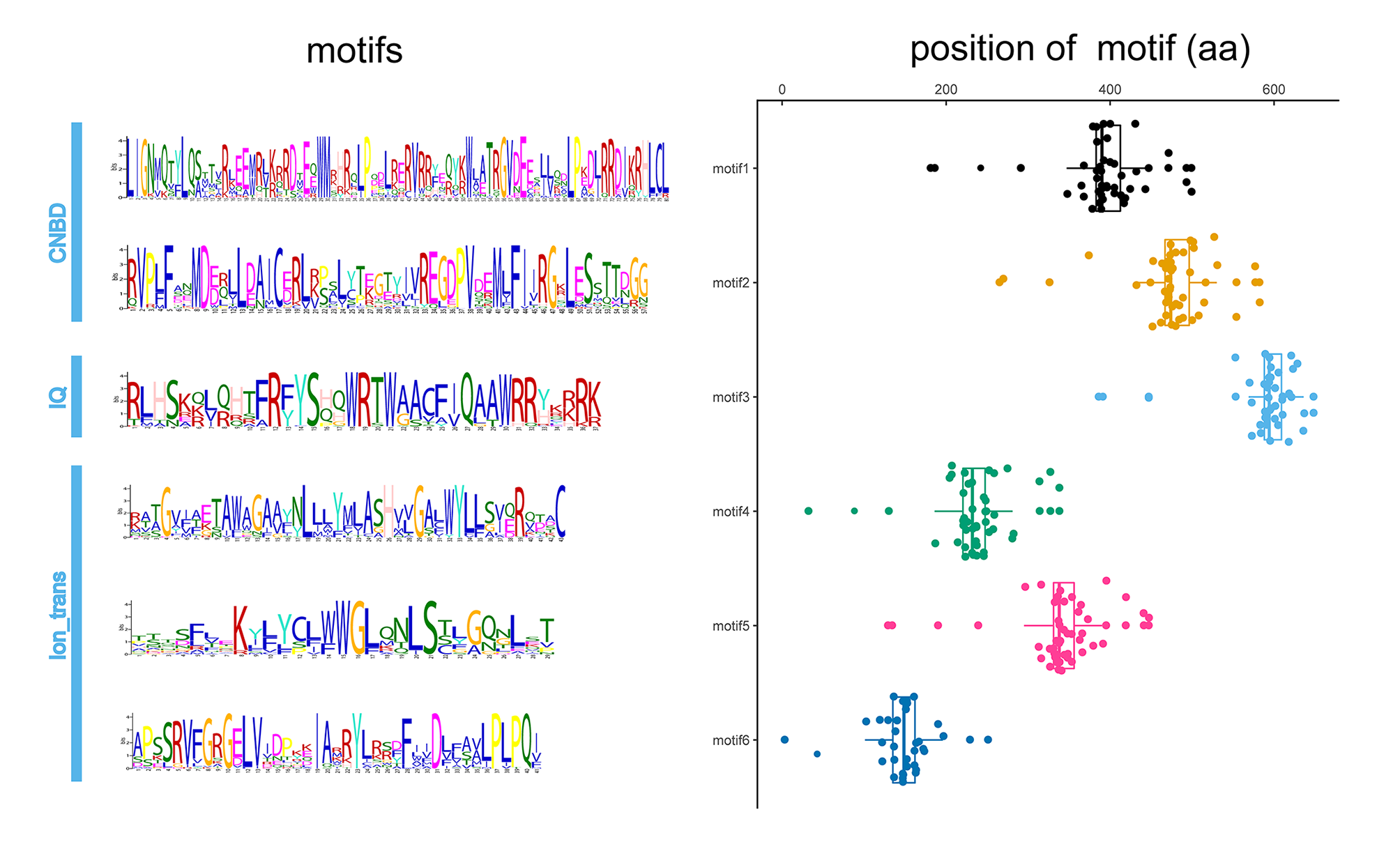

Supplement: Figure S2 — Motif component and position in the TaCNGC genes. Functional motifs were presented on the right using MEME, and clustered based on function. The position of those motifs performed by the box plots, and x-axis indicated the length of all the TaCNGC genes (aa). [file Image2.TIF]

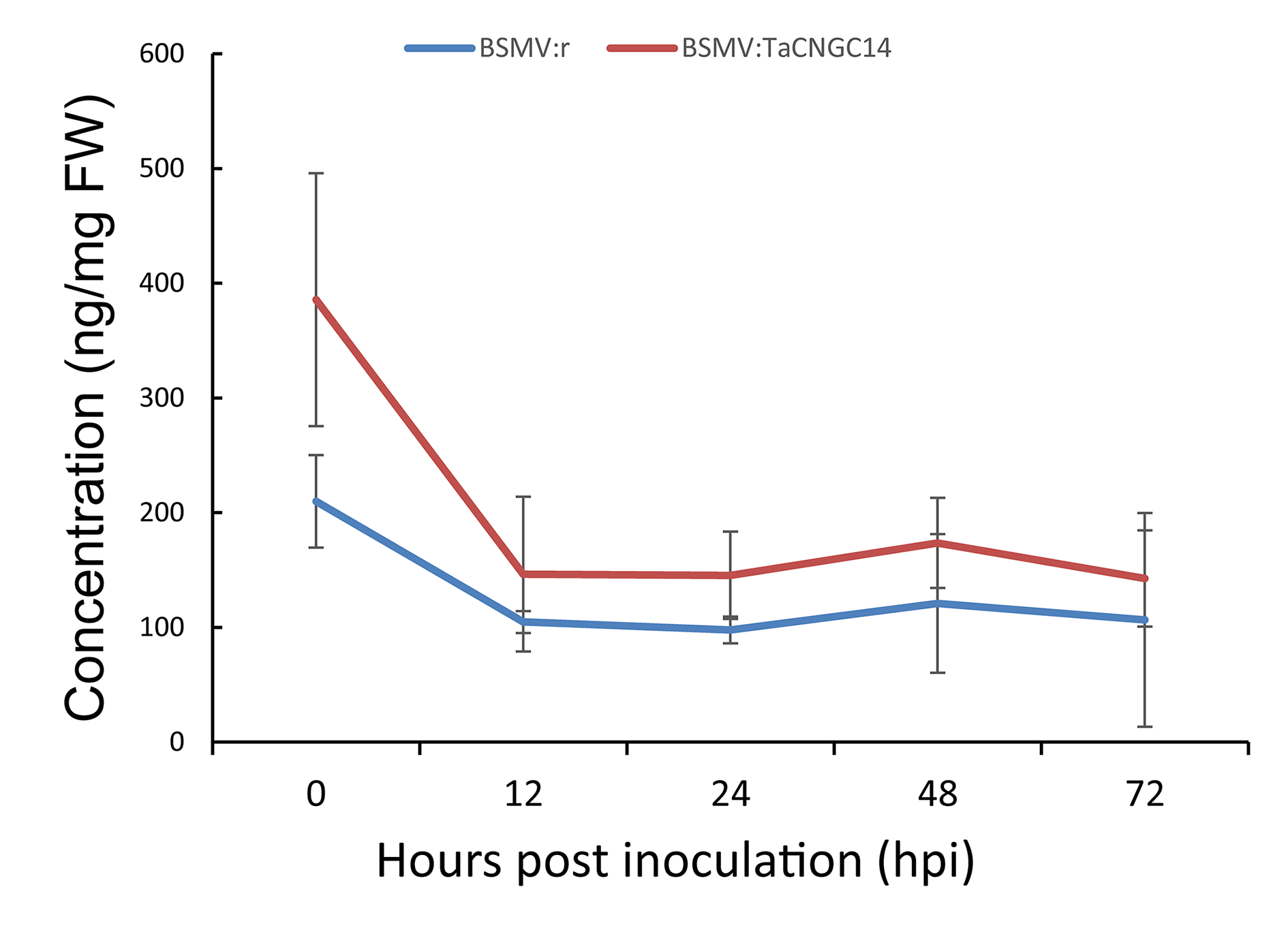

Supplement: Figure S3 — Quantification of SA level in TaCNGC14 silencing plants. Silencing leaves which inoculated with CYR23 were isolated from the fourth leaves, BSMV:γ plant is as a control. Results are shown as means ± SE of three biological replications. [file Image3.TIF]

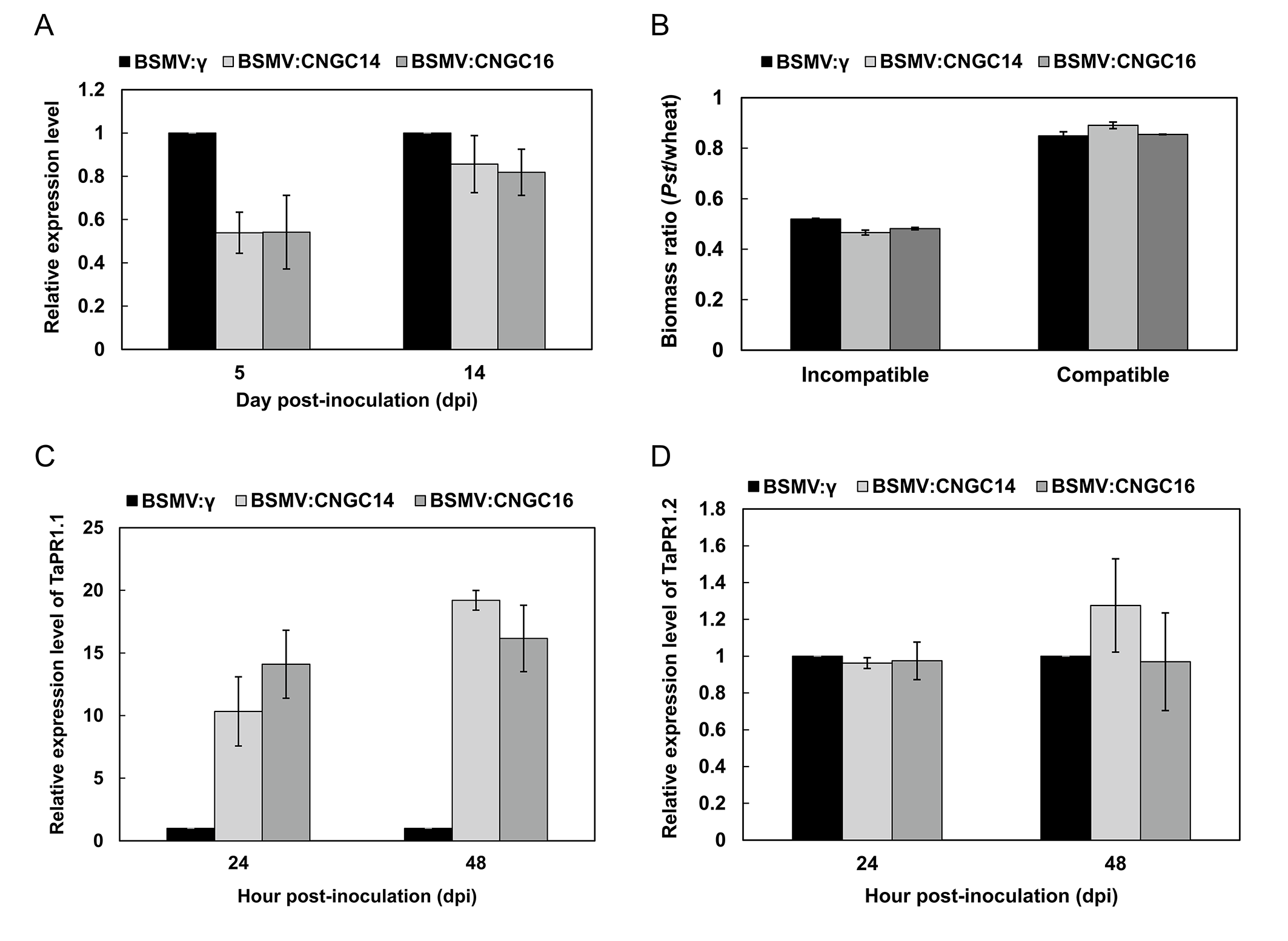

Supplement: Figure S4 — qRT-PCR analysis of TaCNGC14 and TaCNGC16 silencing plants. (A) Silencing efficiency assessment of two TaCNGCs in the fourth leaves of TaCNGCs-knockdown plants inoculated with avirulent race Pst CYR23 at 5 and 14 dpi. (B) Fungal and wheat biomass ratio measured via total DNA content at 14 dpi by absolute quantification using the internal reference genes PsEF and TaEF, respectively. (C,D) Transcriptional changes in PR1.1 genes and PR1.2 in TaCNGCs-knockdown wheat seedlings. [file Image4.TIF]
